# Transferrin and antioxidants partly prevented mouse oocyte oxidative damage induced by exposure of cumulus-oocyte complexes to endometrioma fluid

**DOI:** 10.1186/s13048-020-00738-0

**Published:** 2020-11-26

**Authors:** Zi Ren, Jiana Huang, Chuanchuan Zhou, Lei Jia, Manchao Li, Xiaoyan Liang, Haitao Zeng

**Affiliations:** Center for Reproductive Medicine, Sixth Affiliated Hospital, Sun Yat-sen University, Guangzhou, People’s Republic of China

**Keywords:** Endometrioma fluid, Oocyte oxidative damage, Oxidative stress, Transferrin, Antioxidant, Assisted-reproductive technology, Infertility

## Abstract

**Background:**

Exposure of oocytes to the endometrioma fluid has an adverse effect on embryonic quality. To determine whether adding transferrin and antioxidants to culture medium could counteract detrimental effects on mouse cumulus-oocyte complexes (COCs) induced by exposure to endometrioma fluid or not, we conducted an in vitro cross-sectional study using human and mouse COCs.

**Methods:**

Eighteen women who had their oocytes exposed to endometrioma fluid during oocyte retrieval were enrolled. COCs from superovulated ICR female mice were collected. They were first exposed to human endometrioma fluid and then treated by transferrin and/or antioxidants (cysteamine + cystine). Subsequently, COCs function was assessed by molecular methods.

**Results:**

This study observed that human COCs inadvertently exposed to endometrioma fluid in the in vitro fertilization (IVF) group led to a lower good quality embryo rate compared to intracytoplasmic sperm injection (ICSI) group. Exposure of mouse COCs to endometrioma fluid accelerated oocyte oxidative damage, evidenced by significantly reduced CCs viability, defective mitochondrial function, decreased GSH content and increased ROS level, associated with the significantly higher pro-portion of abnormal spindles and lower blastocyst formation (*p* < 0.05, respectively). This damage could be recovered partly by treating COCs with transferrin and antioxidants (cysteamine + cystine).

**Conclusions:**

Transferrin and antioxidants could reduce the oxidative damage caused by COCs exposure to endometrioma fluid. This finding provides a promising new possibility for intervention in the human oocyte oxidative damage process induced by endometrioma fluid during oocyte pick-up.

**Supplementary Information:**

The online version contains supplementary material available at 10.1186/s13048-020-00738-0.

## Background

Endometriosis is defined as the presence of endometrial resembling tissues or cells outside of the uterine cavity [1]. Endometriomas are cystic masses arising from the growth of ectopic endometrial tissues and glands within the ovary [2]. It has been estimated that endometriosis is found in 0.5–5% fertile women and 25–40% infertile women [3], among whom between 17 and 44% of patients have endometrioma [4]. Severe endometriosis (stage III/IV), usually associated with the presence of endometrioma, is related to the unsatisfactory implantation and clinical pregnancy rates of IVF therapy, while the surgical intervention of endometrioma cannot alter the pregnancy outcome of IVF/ICSI [5]. Therefore, considering the potentially detrimental effects of surgery, IVF/ICSI is the first option for more and more infertile patients with endometrioma [6]. Nevertheless, it seems to be a common phenomenon that the cumulus-oocyte complexes (COCs) are contaminated by endometrioma fluid during oocyte retrieval.

Endometrioma fluid is formed by the menstrual flow and the debris with excess iron (Fe) accumulation [7, 8]. Iron is essential for organisms’ biological processes, serving for oxygen transport, energy metabolism, ATP generation, and DNA synthesis and repair [9]. Iron overload causes significant cytotoxic effects on living cells. Cellular labile iron pool (LIP), which is composed of redox-active iron (Fe2+), can generate reactive oxygen species (ROS) via iron-catalysed Haber-Weiss reaction [10], therefore causing ROS related DNA, lipid and protein damage [11]. Imbalance between ROS generation and detoxification results in oxidative stress [9]. Evidence has indicated higher levels of ROS and reduced antioxidant enzymatic activity in follicular fluid of women with endometriomas [12, 13]. A significant increased ROS level in follicular fluid is correlated with poor oocyte and embryo quality and lower fertilization rate [14]. Oxidative stress has been proven to be associated with post-ovulatory oocyte aging and apoptosis of early embryo [15]. Peritoneal fluid from endometriosis has pernicious impacts on early embryo development [16, 17]. Patients in the endometriosis group present significantly lower mature oocyte and fertilization rates [18]. Furthermore, researchers have indicated that contamination of endometriotic contents during oocyte retrieval leads to a significantly lower fertilization rate and pregnancy rate [19]. In vivo experiments also have suggested that endometriotic fluid is detrimental to reproductive performance and subsequent blastocyst development [20].

Recent studies have clearly shown that several chemicals are capable of inhibiting oocyte oxidative damage caused by oxidative stress. Transferrin, widely known as a natural iron chelator, is synthesized predominantly by hepatocytes and maintained in stable levels in serum [21]. The main function of transferrin is binding and transportation of iron in the circulation, thereby preventing the participation of iron in redox reactions. Transferrin has the ability to protect the host against the ROS induced by excess free iron [22]. Adding transferrin in the culture medium can prevent mouse 2-cell block [8] and improve blastocyst percentages [23]. Glutathione (GSH) plays a significant role in all aspects of iron metabolism such as sensing and regulating iron levels, iron transporting, and iron cofactor biosynthesis, which is essential for cellular iron homeostasis [24]. GSH is critical for the maintenance and regulation of the thiol-redox status of the cell, thus protecting the cumulus cell, oocyte, and embryo from oxidative damage, as well as improving cytoplasmic maturation and oocyte competence [25]. GSH levels are positively associated with the number of high-quality embryos. And GSH levels in follicular fluid are significantly decreased in patients with endometriosis [26]. Cysteine is essential for redox homeostasis, being a vital precursor of GSH production [27]. Direct supplementation of cysteine into the medium is impracticable as cysteine is easily oxidized to cystine in the culture medium, which is cytotoxic [28]. Because oocytes are unable to take up cystine directly, cystine is taken up by cumulus cells (CCs) firstly and is then converted into cysteine. After that, cysteine is released to the medium and utilized by the oocytes subsequently. Low molecular weight compounds, such as cysteamine, are capable of enhancing cysteine uptake, therefore increasing GSH synthesis and improving cellular protection mechanisms against oxidative aggressions [29].

In this study, we recorded the IVF outcomes of human COCs contaminated by ovarian endometrioma fluid during oocyte retrieval. Subsequently, we explored the effects of endometrioma fluid on mouse COCs, and whether such effects could be counteracted by adding transferrin and cysteamine/cystine to the culture medium or not.

## Methods

### Human ovarian stimulation, oocyte retrieval, and IVF

Thirteen women (mean age 31.5 years) in IVF cycles and five women (mean age 30.9 years) in the ICSI cycles aged from 27 to 35 years old were enrolled, who had their ovarian endometrioma fluid accidentally aspirated during oocyte retrieval from July 2017 to August 2018. They had normal ovarian hyperstimulation with a long protocol or short protocol of GnRH agonist. Thirteen patients of IVF group and four patients of ICSI group were stimulated with long protocol, and one patient was stimulated with short protocol of GnRH agonist. There was no significant difference in fertility rate, high-quality embryonic rate, and pregnancy rate between different stimulation protocols (See Table S1 in supplementary material). When at least two leading follicles reached a mean diameter of 18 mm, 250 μg of HCG (Ovidrel, Serono) was given. COCs were retrieved 35–36 h later by insertion of an oocyte collection needle attached to a 10-ml tube under the guidance of vaginal ultrasound. This was a self-controlled study, and each patient had oocytes with endometrioma fluid contamination or without contamination. Endometrioma fluid contaminated oocytes were placed in a separated culture dish during oocyte retrieval process. Once the needle was contaminated, a new needle was replaced or the previous needle was carefully washed before further use. And then, these COCs were transferred to modified human tubal fluid (mod-HTF) medium with 10% serum protein substitute (SPS, SAGE, USA) and incubated at 37 °C in an atmosphere of 6% CO2 for 2–4 h before insemination. Fertilization was evaluated 16 to 18 h post-insemination. Zygotes with two pronuclei and two polar bodies were recorded as fertilized. Embryos were graded 72 h post-insemination [30]. Informed consent was obtained from all individual participants included in the study. All procedures performed in studies involving human participants were in accordance with the ethical standards (Reproductive Medicine Ethics Committee of The Sixth Affiliated Hospital of Sun Yat-sen University +2017ZSLYEC-013S).

### Animals and reagents

All applicable international, national, and institutional guidelines for the care and use of animals were followed. The use and handling procedures of mice in our study were approved by the Committee on the Use of Live Animals for Teaching and Research of Sun Yat-sen University and Institutional review board. Four-week-old ICR female mice were housed in a temperature-controlled and light-controlled room. Chemicals and reagents were purchased from Sigma (St. Louis, MO) unless otherwise stated. Endometrioma fluid was obtained from human at the time of laparoscopy. The fluid was kept refrigerated under − 80 °C and warmed to 37 °C before the experiment.

### Mouse oocyte recovery

Four-week-old ICR female mice were superovulated with equine chorionic gonadotropin (eCG; 5 IU i.p.), followed by human chorionic gonadotropin (hCG; 5 IU i.p.) 48 h later. Approximately 14 h after hCG injection, the mice were sacrificed by cervical vertebrae dislocation. The oviducts were removed and torn to release COCs, which were subsequently divided into four groups.

### Exposure of COCs to endometrioma fluid and different treatments afterward

In the control group, COCs were transferred into M2 medium. After 15 min, they were washed six times in mod-HTF before being transferred into mod-HTF for culture for 6 hours. In the other three groups, COCs were all first exposed to M2 medium with 10% (v/v) endometrioma fluid for 15 min and then were also washed six times in mod-HTF. The endometrioma fluid was aspirated aseptically from a patient diagnosed as endometrioma during laparoscopic operation without blood contaminated. It was stored at − 80 °C. When the experiment was performed, the endometrioma fluid was thawed in a 37 °C-water bath. Different treatments were done with culture medium for 6 hours of culture afterward. In the second group, COCs were kept in mod-HTF. COCs in the third group were placed into mod-HTF with the addition of bovine transferrin (1 mg/ml). In the fourth group, COCs were transferred into mod-HTF supplemented with Cysteamine (100 μM) and Cystine (200 μM), according to the study of Zhou et al. [31].

### Mouse oocytes IVF

IVF was performed as previously described [32]. Briefly, epididymides from proven fertile male mice were punctured, and the sperm squeezed into medium and allowed to disperse in mod-HTF for 60 min. Motile sperm (0.5 × 10^6^) was added to mod-HTF. Fertilization was assessed 6 hours post-insemination. Fertilized oocytes were cultured in mod-HTF at 37 °C in 5% CO2. The morphology of embryos was evaluated under an inverted phase contrasted microscope (Nikon Diaphot, Japan).

### Differential staining of mouse blastocyst

On the 5th day of embryo culture, blastocysts and hatching blastocysts were subjected to a differential staining protocol for the identification of cells within the inner cell mass (ICM) and trophectoderm (TE) layers [33]. Blastocysts were incubated in 0.5% pronase in morpholino propane sulphonic acid (MOPS) for 2–3 min at 37 °C to remove zona pellucida. Before next each step, blastocysts were washed in MOPS (no protein) unless otherwise stated. Blastocysts were incubated in 10 mM trinitrobenzenesulphonic acid (TNBS) acid in MOPS with 0.4% polyvinylpyrrolidone in the dark at 4 °C for 10 min. Then they were kept in MOPS containing 0.1 mg/ml of anti-DNP (ICN ImmunoBiologicals) at 37 °C for another 10 min. Subsequently, they were placed in MOPS supplemented with a 10% complement (Guinea pig serum; ICN) and 25 mg/ml PI at 37 °C in the dark. After 5 min, blastocysts were transferred to ethanol containing 25 mg/ml Hochest and kept at 4 °C in the dark overnight. The following morning blastocysts were washed thoroughly in ethanol and mounted in glycerol on siliconized slides. Blastocysts were analysed under UV light, and the cell nuclei were counted.

### Embryo transfer

Six to twelve-week-old Swiss female mice were mated with vasectomized males, and six blastocyst stage embryos were surgically transferred to each uterine horn on Day 3.5 of pregnancy under anaesthesia with 2% Avertin (0.015 ml/g body weight) prior to embryo transfer [34]. Each recipient was randomly allocated to receive control embryos in one uterine horn and treatment embryos in the other. The ability of the embryo to establish a pregnancy was assessed on Day 15 of pregnancy, and the number of the fetus was recorded, along with fetal and placental measurements.

### Intracellular GSH assay

Oocyte GSH content was measured according to Luciano et al. [35]. COCs were denuded of cumulus cells. For each sample preparation, 50 denuded oocytes were pooled together in one tube, followed by snap-freeze and stored at − 80 °C for later analysis. Standards containing from 0 to 200 pmol of GSH were prepared simultaneously. A volume of 50 μl of each sample and standard was added in a 96-well microtiter plate. The reaction mixture was freshly prepared with 0.15 mM of DTNB, 0.2 mM of NADPH, and 1.0 IU of GSH reductase/ml in 0.1 M phosphate buffer supplemented with 1 mM of EDTA (pH 7.8). Immediately, 0.1 ml was pipetted in each well, and the plate was analysed at 405 nm in a microtiter plate reader (FlexStation 3, Molecular Devices, CA, USA).

### Intracellular ATP and ADP assay

To determine ADP/ATP levels within MII oocytes and cumulus cells, COCs were denuded of cumulus cells. For each sample preparation, ten oocytes or cumulus cells from 50 COCs from each treatment group were collected in 10 μl of ice-cold distilled water, followed by being snap-frozen and stored at − 80 °C for later analysis. Intracellular ADP/ATP ratios were determined by using a method described by Vesce et al. [36]. Cells were first lysed in low KCl before being assayed for ATP. Pyruvate kinase (2 units/assay) was then added to the buffer. The increase in chemiluminescence (Calbiochem) was recorded in a microtiter plate reader (SpectraCount, Packard, Meriden, CT, USA). Once the ATP signal was detected, 0.5 mM phosphoenol pyruvate was added, and a further increase in chemiluminescence due to the conversion of ADP to ATP was determined.

### Detection of mitochondrial membrane polarization

The practice was performed as we previously described [37]. A concentration of 2 μmol/L and 25 min of JC-1(Molecular Probes, Eugene, OR) staining were used for COCs. The fluorescence was observed by using an LSM-510 confocal laser-scanning microscope (Zeiss, Oberkochen, Germany).

### Measurement of ROS in oocytes

The ROS contents of oocytes were measured by the method reported by Nabenishi et al. [38]. Briefly, the denuded oocytes were transferred to 50 μl droplets of 10 μM 2′,7′-dichlorodihydrofluorescein diacetate (DCHF-DA; Molecular Probe, USA) in PBS for 30 min at 37 °C. After being washed three times with PBS, they were placed onto a glass slide and observed under LSM-510 confocal laser scanning microscope using excitation and emission wavelengths of 502 and 523 nm, respectively.

### Assessment of CCs viability

COCs were denuded by 0.1% hyaluronidase medium and mechanically pipetting. CCs were washed in the holding medium and transferred into separate 30 μl droplet of maturation medium. Subsequently, 30 μl of 0.4% trypan blue solution was added. The viable CCs were counted using a Neubauer counting chamber.

### Microtubules and chromosomes staining

Staining and evaluation of spindles were performed by techniques described elsewhere [39]. For microtubule staining, oocytes were fixed in 2% formaldehyde and 0.2% Triton X-100 in PBS for 30 min, then incubated in anti-tubulin monoclonal antibody (1:300; Sigma-Aldrich, St. Louis, MO) for 60 min, followed by incubation in fluorescein isothiocyanate (FITC)–labeled antimouse antibody (1:50; Sigma-Aldrich) for 30 min. For chromosome staining, oocytes were incubated in propidium iodide (PI, 10 mg/mL; Sigma-Aldrich) for 15 min. Microtubule distribution and chromosome alignment were observed by confocal microscopy (Leica Lasertechnik, Heidelberg, Germany).

Spindle morphology was classified as normal when a barrel-shaped structure with slightly pointed poles formed by organized microtubules was observed and when chromosomes were arranged in a compact metaphase plate at the equator of the spindle. Spindle structure was recorded as abnormal when there was a reduction in the longitudinal dimension of the spindle or when there was complete absence or remnant of dispersing spindle and when chromosomes were displaced from the plane of the metaphase plate or were in condensed appearance.

### Data analysis

Proportional data (embryo development and spindle morphology) were arcsine transformed and analyzed with ANOVA. A Duncan multiple-comparison test was used to locate differences. The software used was the Statistics Package for Social Science (SPSS, Inc.). Data were expressed as the mean ± SD, and *p* < 0.05 was considered to be significant.

## Results

### Developmental competence of human COCs previously exposed to endometrioma fluid

The outcomes of IVF and ICSI were shown in Table 1. In the IVF group, exposure of COCs to endometrioma fluid did not show a significant decrease in fertilization rate (*p* > 0.05) or cleavage rate (*p* > 0.05). However, it resulted in a significantly lower good quality embryo rate (*p* < 0.05). In the ICSI group, there was a slight but non-significant decrease in the developmental competence of COCs that were exposed to endometrioma fluid, compared to the control group (*p* > 0.05). All eighteen women underwent fresh embryo transfer. As a rule, good quality embryos derived from non-contaminated COCs had priority for transfer over those from COCs exposed to endometrioma fluid in the same patient. Nine exposed human embryos of IVF group were transferred. Among them, five exposed human embryos had 20% (1/5) implantation rate, and the other four embryos were cultured for blastocyst embryo with none formation. Twenty-one none- exposed human embryos of IVF group were transferred with implantation rate 33.3% (4/12), and the other seventeen embryos were cultured for blastocyst embryo with nine blastocyst embryos formation. Seven patients got clinical pregnant, achieving six physically healthy babies.

**Table 1:** Outcome of human COCs previously exposed to endometrioma fluid

	IVF		ICSI	
Endometrioma fluid	+	–	+	–
Retrieved oocyte (n)	42	63	17	23
MII oocyte (n)	–	–	16	21
Fertilization rate (%)	27 (64.3) ^a^	46 (73.0) ^a^	12 (75.0) ^a^	16 (76.2) ^a^
Cleavage rate (%)	22 (81.5) ^a^	41 (89.1) ^a^	11 (91.7) ^a^	15 (93.8) ^a^
Good quality embryos rate (%)	11 (26.2) ^a^	29 (46.0) ^b^	7 (41.2) ^b^	11 (47.8) ^b^

Within each row, data with different superscripts are different at *p* < 0.05

### Effects of endometrioma fluid, transferrin, cysteamine and cystine on mitochondrial metabolism and oxidative stress of mouse oocytes

**Fig. 1:**
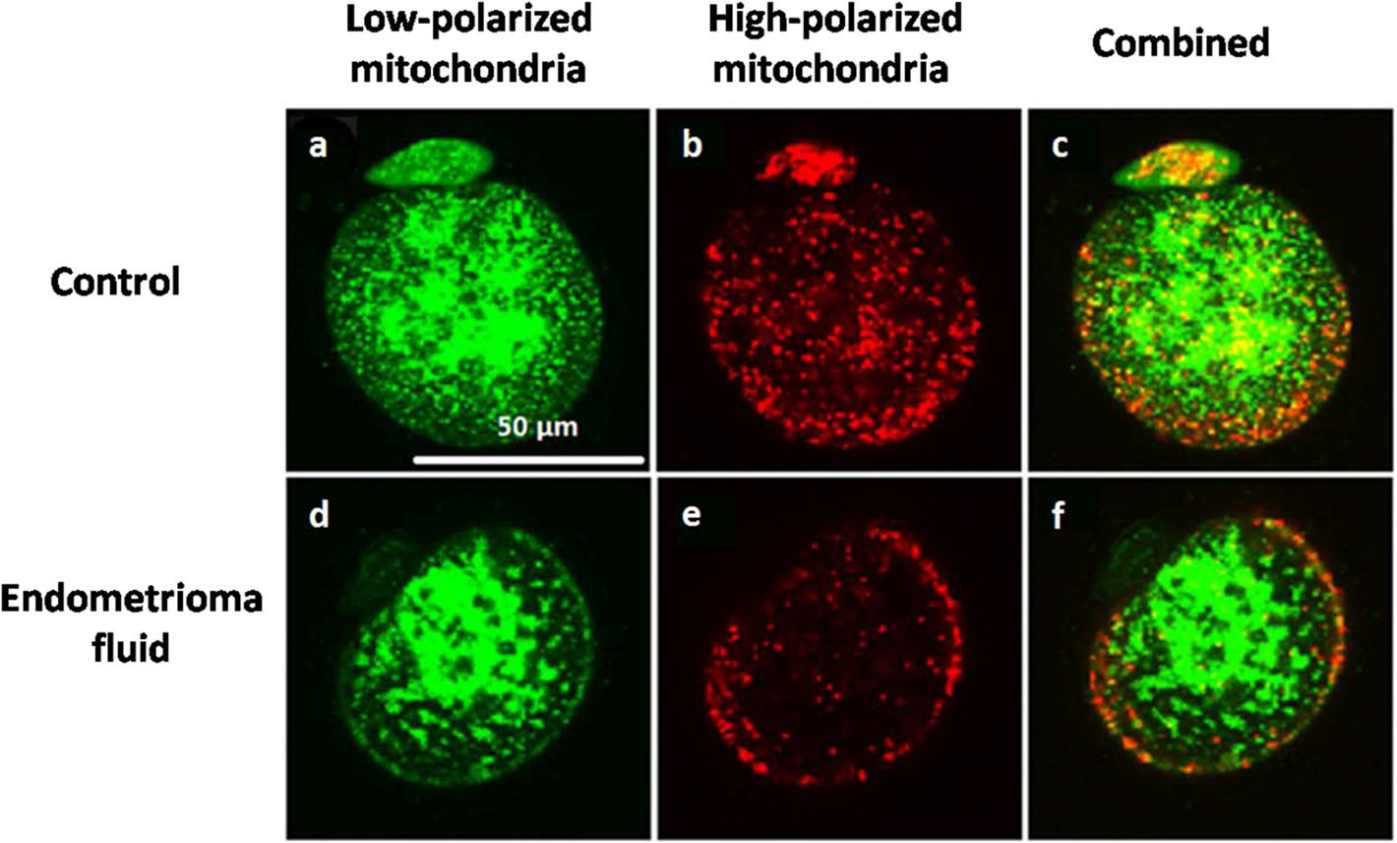
Effects of endometrioma fluid on mitochondrial polarization of mouse oocytes. COCs were denuded and stained for mitochondrial function. **a**, **d** Green fluorescence indicates low polarized mitochondria (δΨm ≤ − 100 mV). **b**, **e** Red fluorescence indicates high polarized mitochondria (δΨm ≥ − 140 mV)

A total of 397 mice were used, of which 290 were four-week-old female mice, 80 were female adult mice, and 27 were male adult mice. The description below the table indicates the number of COCs in each group in each experiment and the number of experiment repeats. Exposure to endometrioma fluid (group 2) versus control (group 1) significantly decreased the mitochondrial membrane polarization (*p* < 0.05) (Fig. 1). Such exposure also induced a drastic decrease in the ATP level (*p* < 0.05), resulting in a significant augmentation in ADP/ATP ratio (*p* < 0.05) (Table 2). When compared with group 2 in which no treatment was applied on those contaminated COCs, culture with transferrin (group 3) led to a statistical increase in ATP level and a significant reduction in ADP/ATP ratio (*p* < 0.05) along with higher mitochondrial membrane polarization (*p* < 0.05). However, treatment with cysteamine and cysteine (group 4) did not statistically alter mitochondrial membrane polarization and ATP level of oocytes (Table 2).

**Table 2:** Effects of endometrioma fluid, transferrin, cysteamine and cystine on mitochondrial metabolism of mouse oocytes

Groups	ATP content (pmol/oocyte)	ADP content (pmol/oocyte)	ADP/ATP	Mitochondrial membrane polarization
1:Control	0.47 ± 0.18 ^a^	0.13 ± 0.05 ^a^	0.28 ± 0.11 ^a^	0.78 ± 0.36 ^a^
2: Exposure to endometrioma fluid	0.33 ± 0.19 ^c^	0.18 ± 0.09 ^a^	0.57 ± 0.21^c^	0.55 ± 0.32 ^c^
3: Exposure to endometrioma fluid +Transferrin	0.42 ± 0.15 ^a,b^	0.15 ± 0.05 ^a^	0.35 ± 0.15 ^b^	0.81 ± 0.47 ^a^
4: Exposure to endometrioma fluid + Cysteamine and Cystine	0.37 ± 0.22 ^b,c^	0.14 ± 0.03 ^a^	0.38 ± 0.19 ^b^	0.61 ± 0.43 ^c^

Within each column, data with different superscripts are different at *p* < 0.05. Each treatment was repeated four times with 5 oocytes for each time for detection of mitochondrial membrane polarization. To determine ADP/ATP levels within oocytes, 10 oocytes from each treatment were collected for each sample preparation

Compared with control COCs (group 1), COCs exposed to endometrioma fluid (group 2) showed a 63% elevation in ROS level (*p* < 0.05) and a 33.3% reduction in GSH content (*p* < 0.05) (Table 3). The incorporation of transferrin (group 3) or cysteamine and cysteine (group 4) in culture medium both lowered the level of ROS in oocytes from contaminated COCs (*p* < 0.05). Transferrin (group 3) did not significantly have an impact on GSH content in oocytes, while a significant augmentation of GSH content in oocytes was observed when treatment with cysteamine and cysteine (group 4) (*p* < 0.05) (Table 3).

**Table 3:** Effects of endometrioma fluid, transferrin, cysteamine and cystine on GSH, ROS level and spindle morphology of mouse oocytes

Groups	GSH (pmol/oocyte)	ROS (Relative ratio)	Abnormal Spindles (%)
1:Control	3.43 ± 1.17 ^a^	1.00 ± 0.33 ^a^	17.2 ± 3.5 ^a^
2: Exposure to endometrioma fluid	2.29 ± 1.01^b^	1.63 ± 0.57 ^c^	29.6 ± 5.4 ^c^
3: Exposure to endometrioma fluid +Transferrin	2.38 ± 0.95 ^b^	1.21 ± 0.53 ^b^	23.8 ± 5.7 ^b^
4: Exposure to endometrioma fluid + Cysteamine and Cystine	3.15 ± 1.36 ^a^	1.15 ± 0.48 ^a,b^	19.4 ± 4.5 ^a,b^

Within each column, data with different superscripts are different at *p* < 0.05. Each treatment was repeated four times with 5 oocytes for each time for detection of ROS. To determine GSH content within oocytes, each treatment was repeated four times with 5 samples for each time, and 50 oocytes in one sample preparation. To determine the spindle morphology, each treatment was repeated four times with 30 oocytes for each time

### Effects of endometrioma fluid, transferrin, cysteamine and cystine on mouse oocytes spindles

**Fig. 2:**
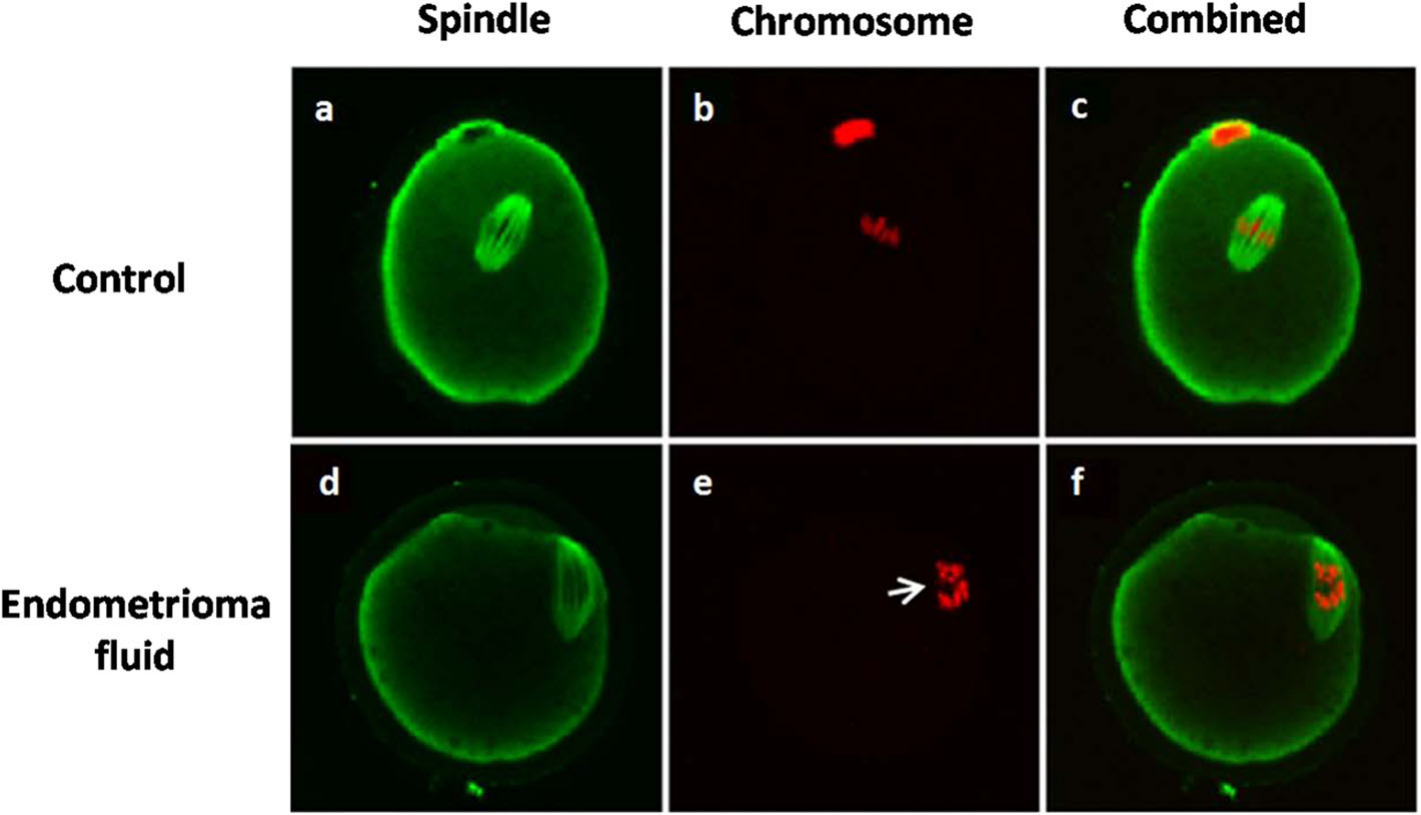
Effects of endometrioma fluid on spindle and chromosome of mouse oocytes. COCs were denuded and stained for mitochondrial function. **a**, **d** Green fluorescence indicates spindle. **b**, **e** Red fluorescence indicates chromosome. **a**, **b** Normal chromosomes and spindles shows barrel-shaped structure with slightly pointed poles formed by organized microtubules with chromosomes arranged on a compact plate at the equator. **d**, **e** Abnormal chromosomes and spindles shows a reduction in the longitudinal dimension of the spindle and chromosomes displaced by the plane of the metaphase plate, which is shown by the arrow

Exposure to endometrioma fluid had a detrimental effect on oocyte spindles with a higher proportion of abnormal microtubules and chromosomes (Table 3, Fig. 2) (*p* < 0.05). And the abnormal spindle formation was reversed either by transferrin (group 3) or cysteamine and cystine (group 4) (Table 3) (*p* < 0.05).

### Effects of endometrioma fluid, transferrin, cysteamine and cystine on mouse CCs viability

Exposure to endometrioma fluid (group 2) led to a statistically decrease in CCs viability (*p* < 0.05), with a significant increase in ADP/ATP ratio (*p* < 0.05) when compared with the control group (Table 4). The introduction of either transferrin (group 3) or cysteamine and cysteine (group 4) in mod-HTF, resulted in the elevation of CCs viability and reduction of ADP/ATP ratio, compared with group 2 (*p* < 0.05) (Table 4).

**Table 4:** Effects of endometrioma fluid, transferrin, cysteamine and cystine on mouse cumulus cells (CCs) viability

Groups	ADP/ATP	CCs viability (%)
1:Control	0.34 ± 0.14 ^a^	82.7 ± 3.8 ^a^
2: Exposure to endometrioma fluid	0.72 ± 0.39 ^c^	51.8 ± 9.4 ^c^
3: Exposure to endometrioma fluid +Transferrin	0.49 ± 0.18 ^b^	80.3 ± 7.7 ^a^
4: Exposure to endometrioma fluid + Cysteamine and Cystine	0.46 ± 0.25 ^b^	70.4 ± 8.2 ^b^

Within each column, data with different superscripts are different at *p* < 0.05. Each treatment was repeated three times with 5 samples for each time. To determine ATP/ADP content within cumulus cells, cumulus cells denuded from 50 COCs from each treatment group were collected for each sample preparation

### Effects of endometrioma fluid, transferrin, cysteamine and cystine on the outcome of mouse IVF and embryo transfer

Exposure of COCs to endometrioma fluid (group 2) significantly reduced blastocyst formation (*p* < 0.05) and total cell number of blastocysts (*p* < 0.05) on day 5 (Table 5). Supplementation of transferrin in mod-HTF (group 3) statistically enhanced blastocyst formation on day 5 (*p* < 0.05) and elevated total cell number of blastocysts (*p* < 0.05) (Table 5). Cysteamine and cysteine (group 4) also assisted in the production of blastocysts with more total cell numbers from COCs exposed to endometrioma fluid (*p* < 0.05) (Table 5).

**Table 5:** Effects of endometrioma fluid, transferrin, cysteamine and cystine on outcome of mouse IVF

Groups	Cleavage (%)	Blastocyst on day 5 (%)	Total cell number of blastocysts	ICM/total cells of blastocyst (%)
1:Control	82.0 ± 7.2 ^a^	67.4 ± 4.3 ^a^	56.3 ± 5.5 ^a^	0.41 ± 0.04 ^a^
2: Exposure to endometrioma fluid	78.8 ± 9.3 ^a^	40.7 ± 6.9 ^c^	46.9 ± 6.1 ^b^	0.38 ± 0.05 ^a^
3: Exposure to endometrioma fluid +Transferrin	76.7 ± 5.7 ^a^	54.3 ± 5.2 ^b^	57.1 ± 6.7 ^a^	0.42 ± 0.05 ^a^
4: Exposure to endometrioma fluid + Cysteamine and Cystine	81.1 ± 7.6 ^a^	49.2 ± 6.6 ^b,c^	53.8 ± 4.7 ^a^	0.39 ± 0.05 ^a^

Within each column, data with different superscripts are different at *p* < 0.05. For total cell number counts, each treatment was repeated five times with 10 blastocysts for each time

Although exposure to endometrioma fluid did not exert an unfavourable influence on implantation rates, fetal survival rates, or placental weight (*p* > 0.05), it significantly reduced fetal weight (*p* < 0.05) (Table 6). Treatment with transferrin (group 3) boosted the fetal weight when compared with group 2 in which no treatment was applied to contaminated COCs (*p* < 0.05) (Table 6).

**Table 6:** Effects of endometrioma fluid, transferrin, cysteamine and cystine on mouse embryo transfer

Groups	Implantation (%)	Fetal development /implantation (%)	Fetal weight (g)	Placental weight (g)
1:Control	72.4 ± 7.8 ^a,b^	44.6 ± 10.5 ^a^	0.82 ± 0.06 ^a^	0.12 ± 0.01 ^a^
2: Exposure to endometrioma fluid	69.2 ± 6.5 ^a,b^	41.7 ± 8.1 ^a^	0.73 ± 0.05 ^b^	0.11 ± 0.01 ^a^
3: Exposure to endometrioma fluid +Transferrin	73.3 ± 9.2 ^a,b^	46.4 ± 7.3 ^a^	0.84 ± 0.06 ^a^	0.11 ± 0.01 ^a^
4: Exposure to endometrioma fluid + Cysteamine and Cystine	67.0 ± 7.2 ^b^	42.9 ± 8.8 ^a^	0.79 ± 0.07 ^a,b^	0.12 ± 0.01 ^a^

Within each column, data with different superscripts are different at *p* < 0.05. Each treatment was repeated three times with 5 Swiss female mice. Six blastocyst stage embryos were surgically transferred to each uterine horn

## Discussion

The impacts of endometrioma fluid on human oocytes quality were contradictory in previous studies. For instance, Khamsi et al. [40] have indicated that endometrioma fluid contamination does not have adverse effects on the fertilization rate (60% vs. 56% in 60 contaminated oocytes and 50 non-contaminated oocytes from 14 patients). Consistently, in mouse model, the fertilization, cleavage and blastocyst formation rates after exposure to endometriotic fluid are not significantly different [20, 41]. Nevertheless, Suwajanakorn et al. [19] have reported a significant decrease in fertilization rates (67% vs. 78% in 85 contaminated oocytes and 301 non-contaminated oocytes in 38 patients). Exposure to endometriotic peritoneal fluid results in the attenuation of oocyte and embryo development [42]. In our present study, we did not find significant alteration of fertilization rate after exposure to endometrioma fluid (75% vs. 76% in 16 contaminated oocytes and 21 non-contaminated oocytes from 5 ICSI patients; 64% vs. 75% in 42 contaminated oocytes and 53 non-contaminated oocytes from 13 IVF patients). On the other hand, a good quality embryo rate was significantly impaired by the contamination of endometriotic contents in the IVF group, but not in the ICSI group. Previous research has elucidated that the attachment of cumulus cells to the oocyte during IVF is vital to promote fertilization and embryonic development [43]. Therefore, the different outcomes between the IVF group and the ICSI group might be attributed to the exposure of cumulus cells to endometrioma fluid. In addition, since the cumulus cells were almost immediately removed after oocyte pick up, it is possible that the presence of CCs “traps” the endometrioma fluid in contact with the oocyte causing the detrimental effects. Therefore, ICSI may be used to avoid the detrimental effects such as poor embryo quality caused by the contamination of endometrioma fluid.

A randomized trial of adequate size is required to further explain the above phenomenon; Therefore, we further constructed a mouse model study, indicating that exposure of COCs to endometrioma fluid accelerated oocyte oxidative damage, evidenced by reduced CCs viability, defective mitochondrial function, decreased GSH content and high ROS level. A variety of factors play essential roles in oocyte oxidative damage including environmental conditions, oocyte interactions with CCs and various chemical components [44]. In particular, oxidative stress is crucial in oocyte oxidative damage by inducing mitochondrial dysfunction and impairing other intracellular components of the oocytes including DNA, proteins, lipids and so on [45]. CCs that surround the oocyte can protect oocytes against the damaging effects of ROS [46]. ROS such as superoxide, H2O2, and HOCl are produced continuously in mitochondria because of the “leakage” of high-energy electrons along the electron transport chain [47]. Mitochondrion is particularly susceptible to oxidative damage, and its regulation of apoptosis is central to cell survival [48]. For example, oxidative stress selectively inhibits mitochondrial respiratory-chain enzymes, thereby decreasing ATP synthesis [49, 50]. ROS oxidizes mitochondrial pores further contributing to cytochrome c release upon GSH depletion [48].

Although a previous meta-analysis has suggested that antioxidants are not correlated with an increased live birth rate or clinical pregnancy rate [51], several antioxidants can prevent chromosome and spindle misalignments and aneuploidy in mouse oocytes and embryos [52]. Recent studies have clearly shown that a number of chemicals possess the capacity to prevent oocyte oxidative damage. Hence, managing oocyte oxidative damage by supplying such chemicals to the culture medium may be particularly critical to improving modern ART technologies [45]. Iron in endometrioma fluid is likely one of the key factors attributing to oocyte oxidative damage. Iron overload displays significant toxicity to the living cell because irons act as progenitors of ROS and molecules that lead to oxidative stress [53]. Oxidative stress is mediated by ROS and results in an imbalance of the intracellular redox potential [54]. Our study elucidated that treating COCs with transferrin could in part restore CCs viability, mitochondrial function, and anti-oxidative stress ability.

In reproduction, GSH is defined as an index of ooplasma maturation, and it participates in sperm decondensation and male pronucleus formation [25, 54, 55]. The incorporation of cystine or cysteamine into the cells is the major limiting step in the synthesis of GSH, which performs a major role in protecting cells from ROS and electrophiles. The present study demonstrated that treating those COCs previously exposed to endometrioma fluid with cysteamine and cystine reduced oxidative stress by increasing GSH level and decreasing ROS level. Meanwhile, cysteamine and cystine supplementation was able to protect the CCs against endometrioma fluid induced mitochondrial dysfunction and subsequent cell death.

A series of morphological and cellular changes occurring during the oocyte oxidative damage process and fertilization of aged oocytes affect not only pre- and post-implantation embryo development but also the later life of the offspring. Functional changes associated with oocyte oxidative damage include decreased fertilization rates, polyspermy, digyny, parthenogenesis, chromosomal anomalies, apoptosis, structural alteration and abnormal and/or retarded development of embryos/fetuses [44]. Although we did not observe the detrimental effects of endometrioma fluid on cleavage rate, implantation rate, fetal survival, and placental weight, blastocyst formation, the total cell number of the blastocysts and fetal weight were significantly lower in those oocytes that were previously exposed to endometrioma fluid. An adverse effect of iron in the development of the mouse embryo in vitro has been found from the 1-cell stage to the blastocyst in a dose-dependent way, with the embryo development being blocked at an early cleavage stage and many of them dying subsequently [8]. In our study, exposure of COCs to endometrioma fluid caused damage to the microtubules and chromosomes, which might be another probably attributing factor for compromised embryo and fetal development. The meiotic spindle plays a critical role in maintaining chromosomal organization. Disorganization of the meiotic spindle can result in chromosomal dispersion, failure of normal fertilization, and abnormal development [56].

This study observed that human COCs inadvertently exposed to endometrioma fluid in the IVF group led to a lower good quality embryo rate. It was supported by further mouse study that exposure of COCs to endometrioma fluid accelerated oocyte oxidative damage via COCs metabolism and anti-oxidative stress ability, with impaired embryonic and fetal developmental competence. This damage could be recovered partly by treating COCs with transferrin and antioxidants (cysteamine + cystine), which provides a promising new possibility for intervention in the human oocyte oxidative damage process induced by endometrioma fluid during oocyte pick-up. However, the limitation of this study was that the ideal controls (exposure of oocytes to human follicular fluid of healthy patients) should have been performed.

## Supplementary Information

**Additional file 1:** **Table S1.** The fertility outcomes of ICSI patients with long protocol and antagonist protocol.

## Data Availability

The datasets used and/or analysed during the current study are available from the corresponding author on reasonable request.

## Abbreviations

COCs: Cumulus-oocyte complexes
IVF: In vitro fertilization
ICSI: Intracytoplasmic sperm injection
LIP: Labile iron pool
ROS: Reactive oxygen species
GSH: Glutathione
CCs: Cumulus cells
mod-HTF: Modified human tubal fluid
SPS: Serum protein substitute
eCG: Equine chorionic gonadotropin
hCG: Human chorionic gonadotropin
ICM: Inner cell mass
TE: Trophectoderm
MOPS: Morpholino propane sulphonic acid
TNBS: Trinitrobenzenesulphonic acid
