# Supplementary material for: Transferrin and antioxidants partly prevented mouse oocyte oxidative damage induced by exposure of cumulus-oocyte complexes to endometrioma fluid
Source: J Ovarian Res. 2020 Nov 26;13:139. doi: 10.1186/s13048-020-00738-0 (PMC7690000; doi:10.1186/s13048-020-00738-0)
Supplement: Supplementary file 1 — Additional file 1: Table S1. The fertility outcomes of ICSI patients with long protocol and antagonist protocol. [file 13048_2020_738_MOESM1_ESM.docx]

Supplementary Material

**Table S1. The fertility outcomes of ICSI patients with long protocol and antagonist protocol.**

| **Protocol** | **Long protocol** | **Antagonist protocol** |
| --- | --- | --- |
| Patients number | 4 | 1 |
| Retrieved oocyte (n) | 31 | 9 |
| Retrieved oocyte (n) | 29 | 8 |
| Fertility rate (%) | 22 (75.9) | 6 (75) |
| Cleavage rate (%) | 20 (90.9) | 6 (100) |
| Good quality embryos rate (%) | 14 (45.2) | 4 (44.4) |

There was no significant difference in fertility rate, high-quality embryonic rate, and pregnancy rate between different stimulation protocols.
